# MPMABP: A CNN and Bi-LSTM-Based Method for Predicting Multi-Activities of Bioactive Peptides

**DOI:** 10.3390/ph15060707

**Published:** 2022-06-03

**Authors:** You Li, Xueyong Li, Yuewu Liu, Yuhua Yao, Guohua Huang

**Affiliations:** 1 School of Electrical Engineering, Shaoyang University, Shaoyang 422000, China; youli9609@163.com (Y.L.); xylihnsyu@163.com (X.L.); 2 College of Information and Intelligence, Hunan Agricultural University, Changsha 410128, China; yuewuliu@whu.edu.cn; 3 School of Mathematics and Statistics, Hainan Normal University, Haikou 571158, China; yaoyuhua2288@163.com

**Keywords:** bioactive peptide, convolution neural network, deep learning, long short-term memory, multi-label issues

## Abstract

Bioactive peptides are typically small functional peptides with 2–20 amino acid residues and play versatile roles in metabolic and biological processes. Bioactive peptides are multi-functional, so it is vastly challenging to accurately detect all their functions simultaneously. We proposed a convolution neural network (CNN) and bi-directional long short-term memory (Bi-LSTM)-based deep learning method (called MPMABP) for recognizing multi-activities of bioactive peptides. The MPMABP stacked five CNNs at different scales, and used the residual network to preserve the information from loss. The empirical results showed that the MPMABP is superior to the state-of-the-art methods. Analysis on the distribution of amino acids indicated that the lysine preferred to appear in the anti-cancer peptide, the leucine in the anti-diabetic peptide, and the proline in the anti-hypertensive peptide. The method and analysis are beneficial to recognize multi-activities of bioactive peptides.

## 1. Introduction

Bioactive peptides are small protein fragments, that generally contains 2–20 amino acid residues [1,2]. The bioactive peptides remain inactive when they are encrypted in the precursor protein, while the bioactive peptides are active once they are released from the precursor protein. Bioactive peptides are not only distributed widely in foods, plants, and animals [3], but also play versatile roles in the metabolic and biological processes. For example, some bioactive peptides were reported to resist the action of digestion peptidases [4], some were proved to be of anti-bacterial and anti-oxidant activity [1], while some had immunomodulatory and anti-cancer activities [5]. Therefore, it is of great importance to accurately identify activities of bioactive peptides in at least two respects: (1) it is helpful to promote understanding of the mechanism of bioactive peptides; and (2) it is fundamental to develop new natural foods and drugs to meet the demands for safety and health.

The bioactive peptides are organic substances comprising amino acids joined by covalent bonds. According to mode of action, the bioactive peptides are classified as the anti-microbial peptide (AMP), the anti-diabetic peptide (ADP), the anti-hypertensive peptide (AHP), the anti-inflammatory peptide (AIP), the anti-cancer peptide (ACP), the anti-oxidant peptide, the immunomodulatory peptide, and so on [3,6]. Since the AMP have anti-bacterial, anti-fungal, or anti-viral properties, they are also called host defense peptides (HDPs) which are distributed widely in the innate immune response system. When the host was invaded by foreigners such as a virus or bacterium, the AMP was induced to destroy or kill the invading foreigners with the membrane damage mechanism [1,7]. The AHP has anti-hypertensive activity by mainly targeting the inhibiting angiotensin-converting enzyme (ACE) which exerts a crucial role through the renin angiotensin system (RAS) in the regulation of blood pressure and electrolyte balance [8]. The AIP is endogenous, and is able to inhibit antigen-specific T(H)1-driven responses [9]. The ACP is able to inhibit tumor cell proliferation or migration, or block the formation of tumor blood vessels, and is less likely to induce drug resistance [10]. The anti-oxidant peptide has anti-oxidant activity, which poses influences on the cells by removing free radicals, inhibiting lipid peroxidation, and interacting with metal ions [11].

Due to importance to food and health of humans, great efforts have been made to develop methods or techniques to identify activities of bioactive peptides over the past decades. Currently, there are two ways: the classical and the bioinformatics approaches [12]. Although the former can accurately identify bioactive peptides, it is too time-consuming, expensive, and labor-intensive. Especially for vast volumes of bioactive peptides, this approach is insufficient for the task. On the contrary, the bioinformatics approaches can remedy the limitation. Hundreds of bioinformatics approaches have been developed to address a wide range of issues in the field of molecular biology [13,14,15,16,17,18], including stress response protein identification [19], RNA modification identification [20,21,22], post-translational protein modification identification [23,24,25,26,27,28], and genomic island detection [18,29,30]. However, these bioinformatics approaches rely heavily on the accumulation of the known samples and the sophisticated design of algorithms. Recently, a large volume of annotated bioactive peptides has been deposited in many public databases, which facilitates greatly development of the bioinformatics approaches. These bioactive peptides databases include the ADP database BioDADPep [31], the BioPepDB [32], the therapeutic peptide database SATPdb [33], the ACPs database CancerPPD [34], the AHPs database AHTPDB [35], the anti-parasitic peptides database ParaPep [36], the database of peptide sequences PepBank [37], the Peptipedia [38], the anti-inflammatory database PreAIP [39], the target-unrelated peptides database TUPDB [40], and the anti-tubercular peptides AntiTbPdb [41]. For example, the BioPep-UWM, a database of bioactive peptides, deposited more than 3000 active peptides [42].

The annotation and the collection of bioactive peptides pave solid material foundations for computationally identifying its activities, while efficiencies depend heavily on design of the features and the learning algorithms. Since the bioactive peptides are made up of the amino acid residues, the information related to composition, the peptide chain, and the hydrophobic/hydrophilic nature of the amino acid would be influential for its activities. To the best of our knowledge, there are no less than 20 bioinformatics approaches for predicting activities of bioactive peptides [43,44,45,46,47,48,49,50,51,52,53]. Khatun et al. [39] developed a random forest-based method (PreAIP) to computationally recognize the AIP, which employed primary sequence as well as evolutionary and structural information. Manavalan et al. [54] presented an extremely randomized tree-based method for anti-tubercular peptides prediction, which utilized only sequence information. Usmani et al. [55] proposed a support-vector-machine-based method AntiTBpred, and Khatun et al. [56] presented a SVM and random-forest-based combination model for anti-tubercular peptides prediction. Zhang et al. [57] developed a classifier-chain-based ensemble learning method for anti-inflammatory peptides prediction. Hasan et al. [58] generated 66 optimal baseline models by combining 11 different encodings and six different classifiers, and then built a representation learning-based method for identifying neuropeptides. The methods for anti-angiogenic peptide prediction include the SVM-based AntiAngioPred [59], the generalized linear model [60], the AntAngioCOOL [61], the random-forest-based TargetAntiAngio [62], the convolution-network-based AAPred-CNN [63]. The PIP-EL [64], the ProInfam [65], and the ProIn-Fuse [66] are three methods for proinflammatory peptide predictions, while the HemoPI [67], the HemoPred [68], and HLPpred-Fuse [52] are three methods for hemolytic peptide prediction. The methods for discriminating between ACPs and non-ACPs include AntiCP [69,70], iACP [71], ACPP [72], iACP-GAEnsC [73], MLACP [74], TargetACP [75], ACPred [76], ACPred-FL [77], ACPred-Fuse [78], ACP-DL [79], and iACP-FSCM [80], while the methods for distinguishing therapeutic peptides from non-therapeutic peptides include PEPred-Suite [81], PTPD [82], PPTPP [83], and PreTP-EL [84]. Most bioinformatics approaches suffered from the small number of bioactive peptides. He et al. [85] pioneered mutual information meta learning to address small samples of bioactive peptides prediction, while Zhang et al. [48] employed the pre-trained natural language model BERT [86] to predict the AMP.

All the previous methods are only suitable for differentiating specific activity of bioactive peptides. In practice, a bioactive peptide might simultaneously consist of multi-activities. Obviously, to computationally identify activities of bioactive peptides is a multi-label and multi-class issue. Recently, Tang et al. [87] presented a convolution neural network (CNN) and gated recurrent unit (GRU)-based deep learning method (called MLBP [87]) for multi-activities of bioactive peptide prediction. This is a promising avenue for identifying actual activities of bioactive peptides. For the deep learning method, the ability to learn a representation would depend on what components it adopted and ways of combining components. The MLBP [87] is a deep learning architecture with three different scale parallel CNNs followed by the GRU [88]. The CNN is the most widely used architecture of neural work especially in the field of image processing, which is capable of characterizing local properties [89,90], while the long short-term memory (LSTM) is a popular architecture to capture semantics in the context of text sequences [91]. The structure of the CNN followed directly by the LSTM would absorb merits of both components. However, the MLBP attached the GRU to three-scale CNNs, which causes multi-scale information loss. In addition, with the increase in the depth of the deep neural network, the original information about sequences would drop seriously. On the basis of the analysis above, we improved the MLBP [87] in two respects. One is that we used multi-branch CNNs, each followed directly by the semantic architecture to improve representation of peptides. The other is to use the residual network architecture to ensure no loss of information about peptides in the forward process. In addition, we replaced the GRU by the Bi-LSTM. The proposed method is abbreviated to MPMABP. The empirical experiments showed that the MPMABP outperformed the MLBP [87].

## 2. Results and Discussion

### 2.1. Optimization of Parameters

**Figure 1:**
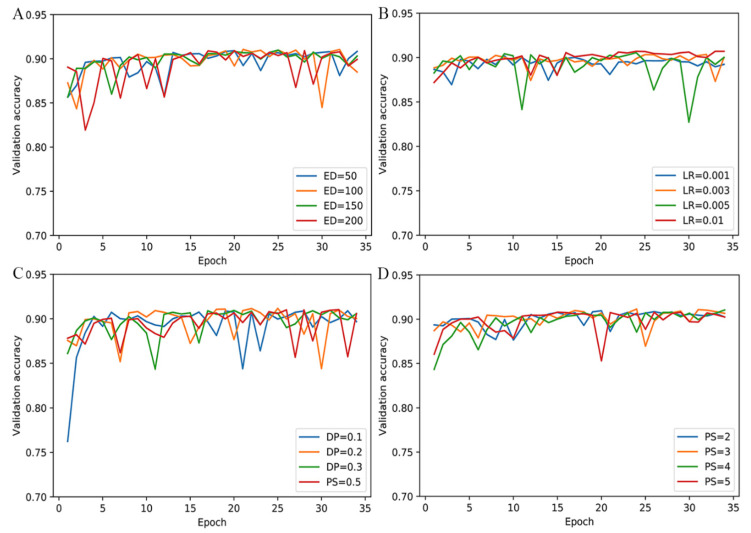
The predictive accuracy of various hyper-parameters. (**A**) ED, (**B**) LR, (**C**) DP, and (**D**) PS denote embedding dimension in the embedding layer, the learning rate, the dropout rate, and the pooling size in the pooling layer, respectively.

In the MPMABP, there are many user-defined hyper-parameters such as the embedding dimension, the learning rate, the dropout, and the pooling size which are influential in its predictive performance. We separated 20 percent from the training set as the validation set to investigate influence. We tested four embedding dimensions (50, 100, 150, and 200), four learning rates (0.1, 0.01, 0.005, 0.003, and 0.001), four dropouts (0.1, 0.2, 0.3, and 0.5), and four pooling sizes (2, 3, 4, and 5). As shown in Figure 1, the accuracies over various values of the same type of hyper-parameter are relatively stable, exhibiting only a slight difference between the various parameter values. According to the general experience, we set the embedding dimension to 100, the learning rate to 0.001, the dropout to 0.5, and the pooling size to 3, respectively. The details of other hyper-parameters in the MPMABP are listed in Table 1.

### 2.2. Comparison with State-of-the-Art Methods

To the best of our knowledge, the MLBP [87] is the latest method to classify multi-functional multi-label bioactive peptides so far. Of course, there are some multi-label algorithms which are applicable to predicting bioactive peptides, such as calibrated label ranking (CLR) [92], random k-label sets (RAKEL) [93], ranking support vector machine, and binary relevance with robust low-rank learning (RBRL) [94], and multi-label learning with deep forest (MLDF) [95]. We conducted the same experiments as the MLBP [87] for comparison. As shown in Table 2, the MPMABP outperformed the MLBP in terms of Precision, Coverage, Accuracy, and Absolute true. The MPMABP promoted the Precision by about 0.034, the Coverage by 0.037, the Accuracy by 0.027, and the Absolute true by 0.011. The lower the Absolute false, the better the predictive performance. The MPMABP decreased the Absolute false by 0.010. We compared five methods over the independent test. As shown in Table 3, we observed the best Precision, the best Coverage, the best Accuracy, the best Absolute true, and the worst Absolute false in the MPMABP, implying that the MPMABP is comprehensively superior to the state-of-the-art methods.

**Figure 2:**
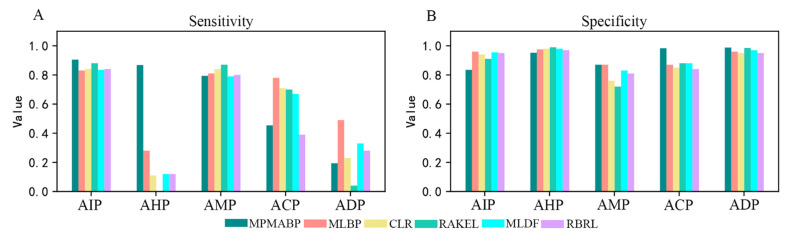
The predictive performance of single-functional bioactive peptides. (**A**) Comparison of MPMABP with other methods on SN, (**B**) Comparison of MPMABP with other methods on SP.

We compared predictive performances of five methods on single functional bioactive peptides. SN and SP were computed by Equations (7) and (8), where the investigated category of bioactive peptides is viewed positive and other as negative. For example, when we computed SN of the AIP, all the AIP bioactive peptides were viewed positive and the other as negative. As shown in Figure 2, predictive performance differs largely with categories. The MPMABP reached the best SN in terms of AIP and AHP, and the best SP in terms of ACP and ADP. However, the MPMABP is inferior to the MLBP in terms of AMP, ACP, and ADP. Since the predictive performances of the MPMABP over AHP are far greater than that of the MLBP, the MPMABP is, as a whole, superior to the MLBP.

Recently, many methods have been developed to identify single activity of bioactive peptides. In order to validate effectiveness and efficiency in classifying activities of bioactive peptides, we compared some state-of-the-art methods which provide web applications, i.e., IAMP-RAAC [96], mAHTPred [97], AHPPred [98], and AIPpred [99]. The IAMP-RAAC [96] is a reduced amino acid cluster-based method for distinguishing AMP from ACP, the mAHTPred [97] is a meta predictor for AHP, the AHPPred [98] is an CNN- and LSTM-based method for AHP prediction, and the AIPpred [99] is a random-forest-based predictor for AIP. Except the IAMP-RAAC [96], all the methods can only be applied to predict specific activity of bioactive peptides. For fair comparison, removing overlapping bioactive peptides with the training samples, we used the overlapping samples with these independent tests in these methods, respectively. Table 4 lists the performances (SN). Obviously, except the mAHTPred, the MPMABP outperformed the other three state-of-the-art methods.

### 2.3. Case Study

In order to prove further predicting ability of the MPMABP, we randomly chose 10 bioactive peptides to be predicted. Table 5 lists predictions by three methods over 10 bioactive peptides. The MPMABP correctly predicted multi-activities of all the bioactive peptides. The MLBP [87] predicted correctly 7, mistakenly 1, and partly correctly 2 of 10 bioactive peptides. MultiPep [100] is also a method which is able to predict up to 12 types of bioactive peptide. We utilized the webserver of the MultiPep: https://agbg.shinyapps.io/MultiPep/ (accessed on 5 February 2022) to perform prediction. Obviously, the MultiPep predicted less classes than the true class for ADP-463 and AIP-1050 and predicted more classes for the other seven bioactive peptides. The 10 cases illustrated the superior predictive performance of the MPMABP over the MLBP [87] and the MultiPep [100].

### 2.4. Discussion

The MPMABP is a CNN and Bi-LSTM-based deep learning method for predicting multi-label bioactive peptides. The MPMABP stacked five CNN and Bi-LSTM modules in a parallel manner. The MPMABP utilized the ResNet to preserve necessary information in the forwarding process. We investigated, respectively, predictive performances of the MPMABPs without the ResNet (called MPMABPwr) and in a series-connection manner (called MPMABPsc). Table 6 shows predictive performances over the 5-fold cross-validation and the independent test. Contrasting Table 6 with Table 2 and Table 3, we found that the inclusion of the ResNet and the parallel manner remarkably improved the predictive performances, respectively.

The CNN and the LSTM are two dominating components in deep learning, each with respective advantages. The CNN is good at characterizing local properties, while the LSTM does well in capturing semantic of words in context of the sequences. We combined two architectures to make full use of their merits. We experimented with many simpler architectures of deep neural network, that is, the MPMABP without the CNN, the MPMABP without the LSTM, and the MPMABP with only one branch. Table 7 and Table 8 show the predictive performance by five-fold cross-validation and by the independent test. The exclusion of the CNN or the LSTM from the original MPMABP lead the predictive performance to decrease. The degeneration of the MPMABP also reduced the ability to accurately classify bioactive peptides

**Figure 3:**
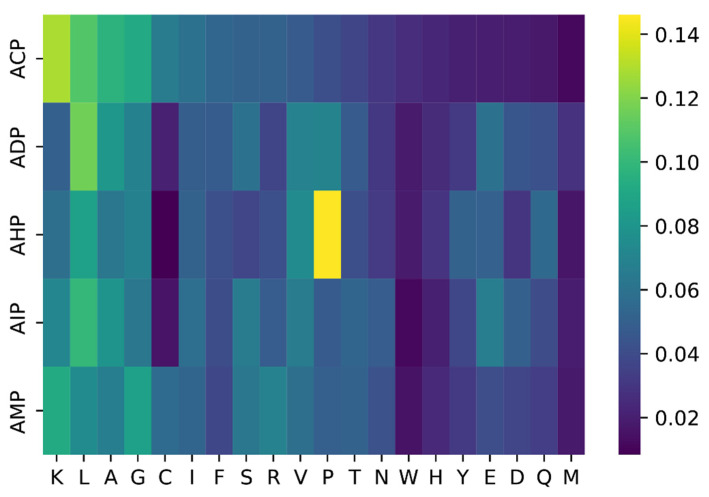
Hot map of amino acid distribution of five types of bioactive peptides.

We investigated distribution of amino acid over five categories of bioactive peptides. As listed in Figure 3, some distributions are common in all classes, but some have remarkable differences across different types of bioactive peptides. The amino acid K appears more frequently in the ACP, P more frequently in the AHP, and L more frequently in the ADP.

## 3. Materials and Methods 

### 3.1. Datasets

**Figure 4:**
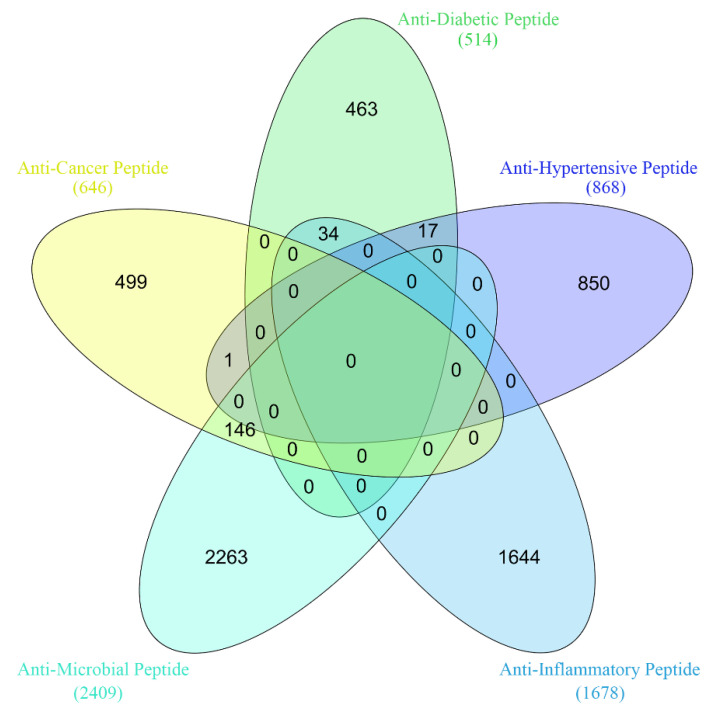
Venn diagram of the dataset.

We used the same experimental dataset as in [87]. The dataset was retrieved by searching the Google Scholar engine with the keyword bioactive peptide, in 2020 [87]. The initial dataset included 18 types of bioactive peptides. Since the number of training samples is too small to train deep neural network favorably, the peptides of less than 500 residues were dropped out. Consequently, five types of functional peptides (AMP, ACP, ADP, AHP, and AIP) were preserved. The clustering tool CD-HIT [101] was used to remove or decrease redundancy and homology. The sequence identity was set to 0.9. The final numbers of the ACP, the ADP, the AHP, the AIP, and the AMP are, respectively, 646, 514, 868, 1678, and 2409, as shown in Figure 4. Obviously, most bioactive peptides are of only one type of activity, a small number of bioactive peptides are simultaneously of two types, and none belong to more than two types. This is a multi-class and multi-label issue. In total, 80 percent of all the peptides were randomly sampled as the training set and the remaining 20 percent were used the testing set.

### 3.2. Methodology

**Figure 5:**
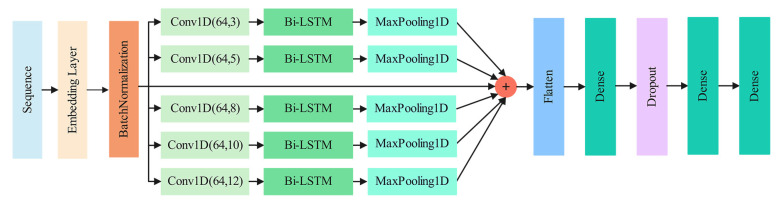
The architecture of the MPMABP. Conv1D represents the CNN layer, MaxPooling1D the pooling layer, and Dense the fully connected layer.

As shown in Figure 5, the proposed MPMABP is an end-to-end deep learning model which is made up of the 1D CNN, the LSTM, the embedding, the batch normalization, and the full-connected layer. The input to the MPMABP is amino acid sequences, which are subsequently transformed into continuous vectors by the embedding layer. Five parallel modules follow the batch-normalization layer to extract deep and abstract representations, of which each is constructed by linking 1D CNN, Bi-LSTM, and max pooling in order. To keep the information, the ResNet structure is used. All the representations are concatenated to be entered into the classification module, which consists of three fully connected layers and a dropout layer. The final fully connected layer has five neurons with the sigmoid function. The output of each neuron stands for a probability of belonging to a corresponding type of peptide.

#### 3.2.1. Embedding Layer

The embedding layer serves as a transformer which converts the sequences of text into continuous digital vectors. Before embedding of text, we pre-processed the peptide sequences. Since the sequence lengths of the bioactive peptides are not identical, ranging from 5 to 517, we padded those peptides of less than 517 residues with the specific character ‘X’. All the characters of peptides were converted into integers. The integer sequences are actually the input to the embedding layer.

#### 3.2.2. Multi-Scale CNN

The CNN is one of most important components for constructing deep complex neural networks, which was initially created by Fukushima et al. [102,103], forming the theoretical foundation by utilizing the backpropagation for training [104], and later was dramatically developed by integration with deep neural networks [89,90,105,106]. At the heart of the CNN is the convolution operation, which is used to multiply receptive fields with the convolution kernel in the element-wise manner and then to sum all the products. The convolution kernel serves as filters in the field of signals, and thus is also called filters. The size of the convolution kernel is influential for representation of the original features. The larger size could capture global structures, while the smaller size could characterize local structure. For extracting different scale representations from sequences, we used five convolution kernels with different sizes. As shown in Figure 4, the smallest size is 3, and the largest size is 12. Therefore, we obtained multi-scale representations of primary sequences.

#### 3.2.3. Bi-LSTM

The LSTM proposed by Hochreiter et al. [107] is an improved recurrent neural network (RNN) [108,109,110]. The LSTM [107] introduces the gate mechanisms such as the forget gate, the output gate, and the input gate, and thus solves well the gradient vanishing or exploding issue occurring in the long sequence analysis. Compared with the traditional RNN, the LSTM is capable of capturing long-distance dependency. Therefore, the LSTM has been used in a wide range of fields including action recognition [111], succinylation prediction [28], and N4-Acetylcytidine prediction [21]. The single LSTM is unidirectional, which is generally able to uncover relationships with previous words. Therefore, the Bi-LSTM is used in practice. The Bi-LSTM [91,112] is composed of two LSTMs in opposite directions, one from the front to the back, and the other from the back to the front. Two LSTMs have identical inputs but have completely different learnable parameters. The outputs of two LSTMs are concatenated as the output of the Bi-LSTM.

#### 3.2.4. Pooling

Pooling is a popular operation in the CNN, which serves as non-linear down-sampling. The pooling has dual roles. One is to decrease the dimensionality of representations, to save storage space, and to accelerate the calculation and another is to avoid over-fitting issues. The pooling operations include max pooling and average pooling. We used the max pooling herein.

### 3.3. ResNet

The ResNet [113] is actually the improved version of the CNN. The ResNet is very simple but effective. As shown in Figure 4, the ResNet consists mainly of two branches: one is to directly link the next layer and the other is the CNN. The sum of the input and the output of the CNN is the output of the ResNet. The ResNet enables construction of deeper neural networks without loss of information. These popular deep learning methods such as VGG, Transformer, and GoogleNet used the ResNet architecture. Here, we used the ResNet to fuse multi-scale representations and original information.

### 3.4. Fully Connected Layer

The fully connected layer is identical to the hidden layers in the multilayer perceptron, which is generally used as linear representations of inputs. Therefore, it is essential to classification or embedding representation in deep learning. We used three fully connected layers, of which the last has five neurons, each to represent a class of the functional peptide. Because this is a multi-label multi-class issue, we used the sigmoid activation function in the last fully connected layer. The neuron outputting more than 0.5 indicated that the input belonged to the corresponding functional peptides. We also used one dropout following the first and the second fully connected layers, respectively, so as to decrease overfitting.

### 3.5. Validation and Evaluation Metrics

We employed both hold-out and 5-fold cross-validation to examine the proposed method. In the hold-out, 80 percent of all the experimental peptides are sampled randomly as the training set, and the remaining 20 percent as the validation set. The model is trained by the training set and then validated by the validation set. In the 5-fold cross-validation, the training set is separated into five parts on average. Four parts are used to train the model and the remaining is used to test the model. The process is repeated five times.

For convenient comparison with the state-of-the-art methods, we used the same evaluation metrics as the MLBP [87], the CLR [92], the Rakel [93], the MLDF [95], and the RBRL [94]. These metrics are defined below.

(1) $$\mathrm{Precision}=\frac{1}{N}\sum_{i=1}^{N}\frac{\left\|L_{i}\cap^{\;}L_{i}^{*}\right\|}{\left\|L_{i}^{*}\right\|}$$

(2) $$\mathrm{Coverage}=\frac{1}{N}\sum_{i=1}^{N}\frac{\left\|L_{i}\cap^{\;}L_{i}^{*}\right\|}{\left\|L_{i}\right\|}$$

(3) $$\mathrm{Accuracy}=\frac{1}{N}\sum_{i=1}^{N}\frac{\left\|L_{i}\cap^{\;}L_{i}^{*}\right\|}{\left\|L_{i}\cup^{\;}L_{i}^{*}\right\|}$$

(4) $$\mathrm{Absolute}\;\mathrm{true}=\frac{1}{N}\sum_{i=1}^{N}ID\left(L_{i},L_{i}^{*}\right)$$

(5) $$\mathrm{Absolute}\;\mathrm{false}=\frac{1}{N}\sum_{i=1}^{N}\frac{\left\|L_{i}\cup^{\;}L_{i}^{*}\|-\|L_{i}\cap^{\;}L_{i}^{*}\right\|}{M}$$

where $L_{i}$ and $L_{i}^{*}$ denote the set of actual labels and predicted labels for the sample I, respectively, *N* is the total number of the testing samples, $\cup^{\;}$ as well as $\cap^{\;}$ denote the union and intersection of the set, respectively, ‖*A*‖ is the number of elements of the set *A*, and ID is defined as:

(6) $$ID\left(L_{i},L_{i}^{*}\right)=\{\begin{array}{cc} 1 & L_{i}==L_{i}^{*}\\ 0 & other \end{array}$$

For Precision, Coverage, Accuracy, and Absolute true, the greater the value meant the better predictive performance. On the contrary, the less Absolute false indicated better predictive performance.

We employed the sensitivity (SN) and specificity (SP) which are the frequently used evaluation metrics in the binary classification. Below are SN and SP definitions:

(7) $$\mathrm{SN}=\frac{TP}{TP+FN}$$

(8) $$\mathrm{SP}=\frac{TN}{TN+FP}$$

where *TP* as well as *TN* are the numbers of the true positive and true negative samples, respectively, and *FP* as well as *FN* are the number of false positive and false negative samples, respectively. This is a multi-label and multi-class issue, not a binary classification. Therefore, we viewed it as five binary classifications. Namely, for a given class, all the samples with such class are positive and others are negative. For example, when we computed SN and SP for the AMP, all the peptides of AMP are positive, and peptides with other classes are negative.

## 4. Conclusions

Most bioactive peptides play therapeutic roles such as resisting microbes and cancer, being potential, safe, and natural organic substances. We presented a CNN and Bi-LSTM deep learning method for classifying multi-label bioactive peptides from the primary protein sequences. Compared with the latest state-of-the-art method (MLBP), the presented method made two remarkable improvements: stacking CNN and Bi-LSTM module in a parallel manner and utilizing the ResNet. The former allows for extracting multi-scale information from sequences, while the latter keeps the information loss lower in the forward process. The inclusion of both improves the predictive performance. We also found that distribution of amino acids varies with category of bioactive peptide. The amino acid P was enriched in the AHP, the L was enriched in the ADP, while the K was enriched in the ACP. The finding is helpful for determining activities of bioactive peptides.

## Figures and Tables

**Table 1:** The details of hyper-parameters in the MPMABP.

Layer	Super-Parameter	Value
Embedding	embedding dimensions	100
CNN layer 1	number of kernels	64
	size of kernels	3
CNN layer 2	number of kernels	64
	size of kernels	5
CNN layer 3	number of kernels	64
	size of kernels	8
CNN layer 4	number of kernels	64
	size of kernels	10
CNN layer 5	number of kernels	64
	size of kernels	12
Pooling layer	size of pooling	3
	stride	1
Bi-LSTM layer	number of neurons	32
Dense1	number of neurons	64
	activation function	relu
Dense2	number of neurons	128
	activation function	relu
Dense3	number of neurons	5
	activation function	relu

**Table 2:** The 5-fold cross-validation results of the training dataset.

Model	Precision	Coverage	Accuracy	Absolute True	Absolute False
MPMABP	0.731 ± 0.011	0.738 ± 0.012	0.722 ± 0.010	0.696 ± 0.013	0.099 ± 0.006
MLBP [87]	0.697 ± 0.012	0.701 ± 0.014	0.695 ± 0.012	0.685 ± 0.011	0.109 ± 0.004

Note: ± indicates standard deviation over the 5-fold cross-validations.

**Table 3:** The independent test results.

Model	Precision	Coverage	Accuracy	Absolute True	Absolute False
MPMABP	0.728	0.749	0.727	0.704	0.101
MLBP [87]	0.710	0.720	0.709	0.697	0.106
CLR [92]	0.667	0.677	0.666	0.655	0.133
RAKEL [93]	0.649	0.648	0.648	0.647	0.141
MLDF [95]	0.649	0.649	0.648	0.646	0.119
RBRL [94]	0.650	0.651	0.649	0.646	0.140

**Table 4:** Comparison with four existing state-of-the-art methods.

	Method	MPMABP	IAMP-RAAC [96]	mAHTPred [97]	AHPPred [98]	AIPpred [99]
Type						
AMP		0.872	0.788	-	-	-
ACP		0.505	0.333	-	-	-
AHP		0.889	-	0.986	0.361	-
AIP		0.914	-	-	-	0.827

**Table 5:** Comparison of MPMABP with two other algorithms by case study.

Sequence	True labels	Prediction		
		MPMABP	MLBP [87]	MultiPep [100]
ACP-499	ACP	ACP	ACP	AMP/anti-virus/ACP/anti-bacterial /anti-fungal
ADP-156	ADP	ADP	ADP	ACE inhibitor/AHP
AHP-665	AHP	AHP	AHP	Neuropeptide/peptidehormone
AIP-1046	AIP	AIP	AIP	AMP/anti-bacterial
AMP-1389	AMP	AMP	AMP	AMP/anti-bacterial
ACP-29	ACP/AMP	ACP/AMP	AMP	ACP/anti-bacterial/anti-fungal
ACP-220	ACP/AMP	ACP/AMP	None	AMP/anti-bacterial/anti-fungal
ADP-463	ADP/AHP	ADP/AHP	ADP	ADP
AIP-1050	AIP/ADP	ADP/AIP	ADP/AHP	ADP
AHP-483	AHP/ACP	AHP	AHP	Antioxidative/ACE inhibitor/AHP

**Table 6:** The predictive performances of MPMABPwr and MPMABPsc.

Model	Precision	Coverage	Accuracy	Absolute True	Absolute False
MPMABPwr ^a^	0.702	0.723	0.701	0.678	0.108
MPMABPwr ^b^	0.697 ± 0.013	0.704 ± 0.022	0.688 ± 0.013	0.663 ± 0.013	0.105 ± 0.003
MPMABPsc ^a^	0.697	0.719	0.696	0.672	0.109
MPMABPsc ^b^	0.704 ± 0.019	0.710 ±0.023	0.694 ± 0.019	0.668 ± 0.018	0.103 ± 0.006

^a^ and ^b^ represent independent test and 5-fold cross-validation, respectively.

**Table 7:** The predictive performance of 5-fold cross-validation.

Model	Precision	Coverage	Accuracy	Absolute True	Absolute False
MPMABP	0.731 ± 0.011	0.738 ± 0.012	0.722 ± 0.010	0.696 ± 0.013	0.099 ± 0.006
No CNN	0.724 ± 0.011	0.729 ± 0.010	0.714 ± 0.011	0.689 ± 0.013	0.101 ± 0.004
No LSTM	0.708 ± 0.017	0.708 ± 0.014	0.698 ± 0.017	0.678 ± 0.020	0.102 ± 0.004
Degeneration	0.725 ± 0.015	0.733 ± 0.015	0.716 ± 0.014	0.688 ± 0.013	0.101 ± 0.009

**Table 8:** The predictive performance of the independent test.

Model	Precision	Coverage	Accuracy	Absolute True	Absolute False
MPMABP	0.728	0.749	0.727	0.704	0.101
No CNN	0.676	0.688	0.675	0.662	0.105
No LSTM	0.659	0.670	0.658	0.645	0.109
Degeneration	0.690	0.708	0.689	0.670	0.111

## Data Availability

Publicly available datasets were analyzed in this study. This data and source code can be found here: https://github.com/Good-Ly/MPMABP (accessed on 21 April 2022).
